# Pregnant Women's experiences with intimate partner violence one year after the COVID‐19 pandemic in Jordan

**DOI:** 10.1002/nop2.1669

**Published:** 2023-02-24

**Authors:** Sanaa Abujilban, Asma’a AbuAbed, Lina Mrayan, Abdulqadir J. Nashwan, Hanan Al‐Modallal, Jalal Damra, Dheaya Alrousan, Shaher Hamaideh

**Affiliations:** ^1^ Department of Maternal, Child and Family Health Nursing, Faculty of Nursing The Hashemite University Zarqa Jordan; ^2^ "National Woman's Health Care Center Amman Jordan; ^3^ Nursing Department Hamad Medical Corporation Doha Qatar; ^4^ Department of Community and Mental Health Nursing, Faculty of Nursing The Hashemite University Zarqa Jordan; ^5^ Department of Nursing Fakeeh College for Medical Sciences Jeddah Saudi Arabia; ^6^ Department of Educational Psychology and Counseling Psychology Hashemite University Zarqa Jordan; ^7^ Faculty of Prince AlHasan Bin Talal The Hashemite University Zarqa Jordan

**Keywords:** COVID‐19, intimate partner violence, Jordan, pregnant

## Abstract

**Aim:**

To assess the prevalence and compare the levels of intimate partner violence (IPV) before and during the pandemic and to identify the factors that associated with physical IPV among Jordanian pregnant women.

**Design:**

A cross‐sectional, correlational design. Women were asked to report their experience with IPV twice: during and before the pandemic.

**Methods:**

A convenience sampling technique was used to select pregnant women from National Woman's Health Care Center from 15 April to 1 September 2021. The Domestic Violence Questionnaire Screening Tool (DVQST) was used to assess the levels of IPV.

**Results:**

The women (*n* = 232) who participated in the study experienced considerable levels of IPV before (69% control IPV, 59.90% psychological, 46.10% physical, 43.10% sexual) and during (75.90% control IPV, 64.20% psychological, 46.10% physical, 40.90% sexual) the pandemic. There were statistically significant (*p* ≤ 0.05) higher mean DVQST scores for control IPV and psychological IPV during the pandemic (control IPV mean = 9.78, psychological mean = 7.03) versus before the pandemic (control IPV mean = 8.95, psychological mean = 6.62). Woman's educational level, marriage duration, woman's employment status, and level of mutual understanding were inversely associated with physical IPV during the pandemic.

**Patient or Public Contribution:**

IPV is a global public health problem and a major violation of human rights. The levels of control IPV and psychological IPV increased during the COVID‐19 pandemic, while the levels of physical and sexual IPV stayed the same. Antenatal screening for IPV is crucial to save women and their offspring from suffering this type of violence.

## 1 INTRODUCTION

Intimate partner violence (IPV) is defined globally as any behaviour by a current or past intimate partner that causes physical, sexual, or psychological harm (World Health Organization, 2021), while for the current study, it refers to any behaviour by husband that causes physical, psychological or sexual harm within a marriage relationship. Intimate partner violence has four forms: Controlling behaviours, including isolating a person from family and friends; monitoring their movements; and restricting access to financial resources, employment, education or medical care. Emotional (psychological) abuse, such as insults, belittling, constant humiliation, intimidation (e.g. destroying things), threats of harm, threats to take away children. Acts of physical violence, such as slapping, hitting, kicking and beating. Sexual violence, including forced sexual intercourse and other forms of sexual coercion (WHO, 2012). This type of violence is considered a global public health problem and a major violation of human rights (World Health Organization, 2021). Globally, one in every three women has been subjected to physical and/or sexual (WHO, 2020b). The rates of IPV differ by geographical region, with Africa having the highest rate (36.10%) and Europe having the lowest (5.10%) (Román‐Gálvez et al., 2021). In the Arab world, women are experiencing substantial levels of IPV (6%–59% physical, 5%–91% emotional/psychological, and 3%–40% sexual) (Elghossain et al., 2019). Furthermore, in Jordan specifically, pregnant women are experiencing considerable levels of all types of IPV (Abujilban, Mrayan, & Damra, 2021). In Jordan, despite the emergency situation caused by the COVID‐19 pandemic, pregnant women are suffering from the violent behaviours of their husbands (80% control, 50.20% psychological, 13% physical, and 11.20% sexual) (Abujilban, Mrayan, Hamaideh, et al., 2021). The latest Jordanian Demographic and Health Survey (DHS) (Department of Statistics (DOS) & ICF, 2019) reported that only 29% of women have never experienced any controlling behaviours by their husbands. Furthermore, the same survey reported that in at least one of the defined situations, 46% of ever‐married women and 69% of all men aged 15 to 49 years old feel that wife beating is justifiable.

Pregnant women constitute a special case, as experiencing IPV affects both the mother and her unborn baby. For instance, mothers who experience IPV are more likely to have prenatal depression (Yu et al., 2018), fewer prenatal visits (Gardner et al., 2012), perinatal death (Pastor‐Moreno et al., 2020), and negative experiences with parenting (Pels et al., 2015). Moreover, foetuses are at risk of low birth weight (Abujilban et al., 2017), preterm birth (Berhanie et al., 2019), birth defects, asphyxia, and stillbirth (Yu et al., 2018).

Since 2019, the world has been suffering from the COVID‐19 pandemic. Jordan is no exception, as at the time of writing this report 1,260,983 cases have been diagnosed with COVID‐19 and 13,248 people have died from COVID‐19 (Worldometers, 2022). COVID‐19 affects everyone, including pregnant women. When a pregnant woman contracts COVID‐19, she is at higher risk of preeclampsia or eclampsia, emergency caesarean birth, and longer hospitalization after birth. Moreover, the baby is at higher risk of fetal death and premature birth (Gurol‐Urganci et al., 2021).

During every form of emergency, including epidemics, violence against women tends to rise (John et al., 2020; WHO, 2020b) because of the risks of loss of income, disruption of social and protective networks, and isolation (WHO, 2020a). In Jordan, the adults who might be most worried about the pandemic are women, and according to them, obtaining gender‐based‐violence services prior to the pandemic was easier than during the lockdown (UNFPA, 2020).

Although there is violence against women in Jordan, and specialists expect this violence to increase during the pandemic (John et al., 2020), there are no published studies showing the impact of the pandemic on violence. In a previous study (Abujilban, Mrayan, Hamaideh, et al., 2021) on a non‐representative sample (online survey) of pregnant Jordanian women, it was found that all types of IPV decreased significantly during the complete lockdown (April to May 2020) in Jordan. However, a year after the pandemic (from 15 April to 1 September 2021), the situation in the country might have changed due to the vast economic and social impacts of the pandemic, and there might have been an increase in the level of IPV. Some pregnant women in Jordan are still suffering from the violent behaviours of their husbands, and only 19% of those who have been survivors of physical or spousal sexual violence have sought help to stop it (Department of Statistics (DOS) & ICF, 2019). This means that more attention needs to be paid to IPV in Jordan in order to understand its antecedents and to put appropriate plans in place to eradicate it. It is for these reasons that this study aimed to assess the prevalence and compare the levels of IPV before and during the pandemic and to identify the factors that associated with physical IPV during the pandemic. To this end, the study sought to answer the following three questions: (1) What are the current levels of IPV? (2) Is there a change in the levels of IPV from before the pandemic? (3) What are the associative factors physical IPV during the pandemic?

The worldwide cost of violence against women was estimated before the pandemic to be around USD 1.5 trillion (UN women, 2020). Knowing levels of IPV during the pandemic and comparing them with the levels before the pandemic could shed light on the exact suffering of pregnant women. Such information could help decision makers in planning the most beneficial tailored protective programs at the appropriate time (e.g., psychological counselling, protection, and social services) (Almeida et al., 2017) in order to overcome IPV. It may also give an indication of the importance of the role of early screening for IPV and highlight ways to help women in emergency situations such as the COVID‐19 pandemic. Moreover, the findings may have broader implications because when people in the community are aware of the scope of the problem, it might be easier to discourage some men from engaging in IPV.

## 2 METHODOLOGY

### 2.1 Design

A cross sectional, correlational design was employed for this study. This design is considered to be the best for researching a variable such as IPV (Polit & Beck, 2011).

### 2.2 Setting

Data were collected from the National Woman's Health Care Center (NWHCC). The center is located in Tafila governorate in the south of Jordan and has a number of clinics. The center aims to provide high‐quality comprehensive care for women of all ages in Tafila province and the other southern governates (Karak and Ma'an) of the country. The center is unique in providing specialized and comprehensive health care. The center has 43 employees, providing reproductive and sexual health services throughout the female lifespan, ranging from the care of newborns, children, and adolescents to the care of women during pregnancy and the menopause, as well as the treatment for various gynaecological diseases. The NWHCC's clinics provide services for 27,000 citizens annually in Tafila, Karak and Ma'an governates (AbuAbed, personal communication, January 4, 2022).

### 2.3 Sample and sampling

All pregnant women in Jordan were considered as the target population, where pregnant women who received care at NWHCC clinics were the accessible population. Women who were married, living with their husband, and pregnant at the time of data collection were eligible for the study. Women who could not read or write in Arabic and those who had a health problem which necessitated hospitalization were excluded from the study. A convenience sampling technique was used to select the study participants, as it was judged to be the most appropriate method to access pregnant women during the pandemic. Thorndike's rule was used to calculate the sample size (10*k + 50), where k equals the number of variables (Thorndike, 1982). To compensate for missing data, the predicted sample size was increased by 10%. As a result, it was calculated that more than 200 individuals were required for the study.

### 2.4 Instrument

The same questionnaire as that used in a previous study (Abujilban, Mrayan, & Damra, 2021) was used in this study. The questionnaire is composed of three self‐report tools:

*Part I: Demographic, Obstetrical, and Gynaecological History* (Clark, Hill, et al., 2009). The Demographic, Obstetrical, and Gynaecological History (DOGH) tool is used to measure the socio‐demographic characteristics of pregnant women. It also measures their obstetrical and gynaecological history. Clark, Hill, et al. (2009) gave their permission to use the instrument for the purposes of the study. In the Jordanian context, the content validity index is 0.83 (Abujilban, Mrayan, & Damra, 2021). The DOGH tool contains 34 questions that use different levels of measurement (nominal, ordinal, or ratio). The DOGH contains the following continuous variables: woman and her husband's age in years, gestational age, marriage duration, woman's personal income, household income, gravity, parity, number of children, compliance to antenatal visits, number of miscarriages, weight, height, and the following categorical variables: woman and her husband's educational level, woman and her husband's employment status, residence place, type of family, relative to husband, type of marriage (monogamy vs polygamy), history of miscarriages, health problems during pregnancy, place of antenatal visits, COVID‐19 information (more details of categorical variables are presented in Table 1). Clark, Hill, et al. (2009) developed the DOGH in English, then they translated it into Arabic and back‐translated it to ensure the accuracy of the translated version.

**TABLE 1:** Demographics and antenatal care.

Item	Subitem	*n*	%
Woman's educational level	Preparatory (grades 7–10)	12	5.2
	Secondary (grades 11 and 12)	88	37.9
	Diploma	62	26.7
	Bachelor's degree	67	28.9
	Master's degree	3	1.3
Woman's employment status	Does not work	187	80.6
	Employed	45	19.4
Residence	Rural	124	53.4
	Urban	108	46.6
Type of family	Nuclear	163	70.3
	Compound	69	29.6
Relative to husband	No	171	73.7
	1st degree relative	16	6.9
	2nd degree relative	30	12.9
	3rd degree relative	15	6.5
Type of marriage	Monogamous	230	99.1
	Polygamous	2	0.9
Husband's employment status	Does not work	9	3.9
	Employed	223	96.1
Husband's educational level	Illiterate	1	0.4
	Primary (grades 1 to 6)	2	0.9
	Preparatory (grades 7 to 10)	14	6.0
	Secondary (grades 11 to 12)	136	58.6
	Diploma	36	15.5
	Bachelor's degree	38	16.4
	Master's degree	4	1.7
	PhD degree	1	0.4
Who decides about your money	Me	8	3.4
	Husband	10	4.3
	Both of us	33	14.2
	Others (e.g. mother and father/ in laws)	1	0.4
Health problem before pregnancy	Epilepsy	1	0.50
	Diabetes mellitus	10	5.40
	Hypertension	12	6.50
	Anaemia	18	9.60
	Vaginitis	73	38.2
	Urinary tract infection	42	22.50
	Heart problem	8	4.30
Health problems during pregnancy	Diabetes mellitus	25	10.8
	Hypertension	33	14.2
	Heart problem	15	6.5
	Twin pregnancy	5	2.2
	Placenta or amniotic problem	11	4.7
	Anaemia	58	25.0
	Vaginitis	139	59.9
	Urinary tract infection	68	29.3
Place of antenatal visits	Public hospital	99	42.7
	Private hospital	4	1.7
	Private clinic	59	25.4
	Maternal and child center	69	29.7
Care provider who gives information	Doctor	106	45.7
	Midwife	128	55.2
	Nurse	73	31.5
	Others	14	6.0
How care provider gives information	Antenatal visit	184	79.3
	Telephone	6	2.6
	Social media, WhatsApp	15	6.5
	Others	3	1.3

*Part II: Domestic Violence Questionnaire Screening Tool* (WHO, 2005). Women's experiences with IPV were measured by using the Domestic Violence Questionnaire Screening Tool (DVQST). This tool was developed by the WHO, and later Clark, Hill, et al. (2009) translated, back‐translated, and validated it for Jordanian culture. The DVQST measures four types of IPV: control behaviours (10 items), psychological (8 items), physical (6 items), and sexual IPV (2 items). Each item has four possible options: never (score 1), once (score 2), little (score 3), and too much (score 4). And items were summed up to have total scores for each of the four subscales. If the participant responds “once,” “a little,” or “too much” for any of the listed violent behaviours, she is considered to be a survivor of IPV and is given the code “1”; otherwise “0” (Clark, Bloom, et al., 2009; Garcia‐Moreno et al., 2006). Examples of IPV items include; tries to keep you from seeing your friends (control behaviour), insulted you or made you feel bad about yourself (psychological IPV), slapped you or threw something at you that could hurt you (physical IPV), and physically force you to have sexual intercourse when you did not want to (sexual IPV).Women were asked to respond twice to the DVQT; the first response about her experience in the last year (during the pandemic) and the second about her experience with IPV before the last year (before the pandemic). The DVQST is a reliable measure for IPV in the Jordanian context, as indicated by a Cronbach's alpha of 0.81(Abujilban, Mrayan, & Damra, 2021).

*Part III: Justification for Wife Beating, Mutual Understanding, and Verbal Fighting Scales* (Clark, Hill, et al., 2009; Yoshikawa et al., 2014). The Justification of Wife Beating Scale was adapted to Jordanian culture by (Clark, Hill, et al., 2009). The scale is a reliable measure (Cronbach's alpha = 0.75) and is composed of six situations. Each situation requires a “yes” or “no” response. If the participant responds “yes” for any of the listed situations, she is considered to show acceptance of wife beating and is coded as “1”; otherwise “0”. The Mutual Understanding Scale and the Verbal Fighting Scale are each assessed by using one rating item on a five‐point scale ranging from 1 = never to 5 = always (Clark, Bloom, et al., 2009). The Mutual Understanding and the Verbal Fighting Scales have previously been validated by eight experts for the Jordanian context, where the content validity index was 0.84 (Abujilban, Mrayan, & Damra, 2021).

### 2.5 Data collection procedures

The data were collected after receiving approval from the University's Institutional Review Board (IRB) and permission from the NWHCC to access the participants. The data were collected by seven research assistants (RAs). The RAs were trained in how to select, motivate, and recruit eligible women in order to decrease experimenter bias. The purposes of the study were fully disclosed, and the rights of the participants were explained to the eligible women. Face‐to‐face recruitment was used to decrease the refusal rate. Women were approached to participate at their most convenient time, that is, while they were waiting in the clinic, in order to increase the response rate. They completed the questionnaire in a quiet room in the NWHCC center. Twenty minutes were needed to fill in the questionnaire. As IPV is a sensitive issue for Jordanian women, they were given privacy while filling in the questionnaire to enable them to be as truthful as possible and to decrease social desirability bias. To protect the privacy of the women involved, each questionnaire was given a number. Data were collected during the pandemic from 15 April to 1 September 2021.

### 2.6 Data analysis

The data were analysed by using the Statistical Package for the Social Sciences version 22. The characteristics of the participants and the types of IPV experienced were described using descriptive statistics (mean and frequency). To compare IPV levels before (before March 2021) and during the pandemic (from March 2021 to April 2022), a paired‐samples t‐test was used. The factors that associated with physical IPV were identified by using multiple linear regression. The estimates that are calculated for the multiple linear regression are the unstandardized regression coefficients (B), the standardized regression coefficients (β), the semi‐partial correlations (sr2), the R, R2, and adjusted R2. Variables which were significantly correlated with the physical IPV and mentioned in the literature to be related to the physical IPV (Al‐Badayneh, 2012; Doi et al., 2019; Shwartz et al., 2020) were inserted into the regression model. Preliminary analyses were done to ensure the appropriateness of the data for analysis, where the findings revealed that there were no violations of the statistical tests' assumptions. The level of significance was set at *p < 0.05*.

### 2.7 Ethical consideration

The study protocol was approved by the Hashemite University IRB (#5/5/2020/2021). All the participants signed an anonymous consent form after being assured that their data would be treated confidentially and that only the researchers would have access to such data for research purposes. The women were motivated to participate in the study by knowing that their information would help other women in a similar situation. The women were also told that their participation was completely voluntary, that they could refuse to answer any question, that they could withdraw from the study whenever they wanted, and that doing so would have no impact on their care. The women were assured that their participation would expose them to minimum risks that were the same as those that they could encounter in their daily life. If the participants were identified as being exposed to IPV, they were followed up and referred to a psychiatric doctor if they greed (consent) for that.

## 3 RESULTS

Two hundred and thirty‐two pregnant women participated in the study. Just over half (56.70%) of the women were ≤ 29 years old (M = 29.17, *SD* = 4.08). The mean monthly household income was 529.36 JD (746.64 USD, *SD* = 211.70, 298.59 USD). Most of the women had a secondary school level of education or above (*n* = 220, 94.80%) and were not employed (*n* = 187, 80.60%). The average number of years that the women had spent in education was 13.26 years (*SD* = 2.05). The mean monthly personal income of the working women was 390.69 JD (551.05 USD, *SD* = 140.50 JD, 198.17 USD). Around half of the women were living in rural areas (*n* = 124, 53.40%), while the rest were living in urban areas. The demographic analysis also showed that the mean age of the women's husbands was 34.85 years old (*SD* = 4.60). The vast majority (*n* = 223, 96.10%) of the husbands were employed in the public sector (*n* = 143, 61.60%). Similar to the women, most of the men had completed a secondary level of education or above (*n* = 215, 92.70%).

In regards to the women's family circumstances, all of them were living with their husband, and the mean marriage duration was 6.37 years (*SD* = 3.80). Most of them were living in a nuclear family (*n* = 163, 70.30%). Around a quarter of them were a relative of their husband (*n* = 61, 26.30%). Almost all were in a monogamous marriage (*n* = 230, 99.10%). Only five (2.20%) had been married before.

As regards the women's pregnancy characteristics at the time of the study, the mean gestational age was 25.22 weeks (*SD* = 8.44). The mean haemoglobin level was 10.68 (*SD* = 1.35). The mean gravidity was 3.45 times (*SD* = 1.49) and the mean number of miscarriages was 1.64 (*SD* = 1.74). The women had 2.80 (*SD* = 0.99) live children on average. The majority of the women were healthy before pregnancy (*n* = 207, 89.20) and their pre‐pregnancy body mass index ranged between 16.40 and 41.90 (M = 23.44, *SD* = 3.85). Some women were experiencing health problems during their current pregnancy, such as diabetes mellitus, hypertension, heart problems, placenta or amniotic problems, anaemia, vaginitis, and/or urinary tract infection. Five of the women (2.2%) were having twin pregnancy. Less than half (*n* = 110, 47.40%) were making regular antenatal visits to a clinic with 4.80 (*SD* = 2.20) visits on average, and around two thirds of the women (*n* = 155, 66.80%) had delayed their antenatal visits because of COVID‐19. Many of the participants (*n* = 198, 85.30%) said that they received information about COVID‐19 from their care provider during their antenatal visits, and 95% (*n* = 197) of them said it was beneficial information. See Table 1 for more details.

### 3.1 IPV during and before the COVID‐19 pandemic

The women who participated in this study experienced considerable levels of IPV before (69% control, 59.90% psychological, 46.10% physical, 43.10% sexual) and during (75.90% control, 64.20% psychological, 46.10% physical, 40.90% sexual) the pandemic. The most frequent type of IPV was control and the least frequent was sexual IPV. Both control IPV and psychological IPV increased during the pandemic, while physical IPV remained at the same level, and sexual IPV decreased during the pandemic. Each type of IPV was strongly correlated (*r* = 0.88–0.94) with itself from before to during the pandemic.

A paired‐sample t‐test was used to evaluate the impact of the COVID‐19 pandemic on the women's scores on the DVQST (IPV level). There were statistically significant (*p* ≤ 0.05) higher mean DVQST scores for control IPV and psychological IPV during the pandemic (control mean = 9.78, psychological mean = 7.03) as compared to before the pandemic (control mean = 8.95, psychological mean = 6.62). The eta squared statistic indicated a moderate effect size for control (0.04) and psychological (0.02) IPV. There were no significant differences (*p* > 0.05) in the DVQST scores for physical and sexual violence. For more details, see Table 2.

**TABLE 2:** The prevalence of each type of intimate partner violence (IPV) before and during the COVID‐19 pandemic and the paired‐sample t‐test result for each IPV type.

Type of IPV	Control		Psychological		Physical		Sexual	
Period	During	Before	During	Before	During	Before	During	Before
*n*	176	160	149	139	107	107	95	100
*%*	75.9	69	64.2	59.9	46.1	46.1	40.9	43.1
*M*	9.78	8.95	7.03	6.62	3.56	3.59	1.11	1.11
*SD*	9.27	9.41	7.38	7.34	4.83	4.79	1.59	1.55
*correlation*	0.91 (*p* = 0.000)		0.91 (*p* = 0.000)		0.94 (*p* = 0.000)		0.88 (*p* = 0.000)	
*t*	3.169		1.969		−0.232		−0.084	
*df*	231		231		231		231	
*p*	0.002		0.050		0.817		0.933	
*Eta square*	0.04		0.02					

### 3.2 Description of marital relationship

Most of the employed participants were working in public sector jobs (*n* = 40, 88.90%). Only 20% (*n* = 8) of the women had the full decision‐making power on how to spend their own money (see Table 1). Most of the women (*n* = 177, 76.30%) do not agree with the statement that a good wife obeys her husband even if she disagrees and 66.40% (*n* = 154) do not agree that a family problem should only be discussed with people in the family. Meanwhile, most of the women (*n* = 179, 77.20%) do not agree that if a man mistreats his wife, others outside of the family should intervene. As regards the justification of wife beating, less than half (*n* = 89, 38.40%) of the participants justified wife beating in specific situations, where the highest percentage of agreement was for the situation where a husband finds out that his wife is being unfaithful (29.70%). As for verbal fighting, around half of the women (*n* = 102, 44%) said that they “never” to “seldom” fought with their husbands. In relation to the level of mutual understanding, only 14.70% (*n* = 34) of the women reported that they and their husbands always understood each other. For more details, see Table 3.

**TABLE 3:** Justification of wife beating, level of mutual understanding, and level of verbal fighting

		*n*	%
Acceptable to beat wife if	husband finds out that wife is being unfaithful	69	29.70
	husband suspects that wife is being unfaithful	37	15.90
	wife refuses sex with him	25	10.80
	wife disobeys him	15	6.50
	wife suspects that husband has other girlfriends	8	3.40
	wife does not complete household work to husband's satisfaction	1	0.40
Level of mutual understanding	Never	12	5.20
	Rarely	34	14.70
	Sometimes	85	36.60
	Often	67	28.90
	Always	34	14.70
Level of verbal fighting	Never	38	16.40
	Rarely	64	27.60
	Sometimes	93	40.10
	Often	34	14.70
	Always	3	1.30

### 3.3 Associative factors with physical violence during the COVID‐19 pandemic

A standard multiple regression was performed on physical IPV during the pandemic as the dependent variable and woman's age, woman's educational level, residence, marriage duration, household income, woman's employment status, number of live children, level of mutual understanding and family type on physical IPV as independent variables. These variables were inserted into the regression model because they were significantly correlated with the dependent variable and mentioned in the previous literature as factors that affect physical IPV (Al‐Badayneh, 2012; Doi et al., 2019; Shwartz et al., 2020).

Table 4 displays the correlations between the variables, the unstandardized regression coefficients (B), the standardized regression coefficients (β), the semi‐partial correlations (sr2), the *R*, *R2*, and adjusted *R2*. The R for regression was significantly different from zero: F (9, 232) = 8.91, *p* = 0.000. Only four of the independent variables contributed significantly to the prediction of the physical IPV level: woman's educational level (sr2 = −0.31), marriage duration (sr2 = −0.14), woman's employment status (sr2 = −0.13), and level of mutual understanding (sr2 = −0.15). Among these four variables, woman's educational level makes the strongest unique contribution (−0.37) to explaining physical IPV.

**TABLE 4:** Standard multiple regression of the effect of woman's age, woman's education, residence, marriage duration, household income, woman's employment status, number of live children, mutual understanding, and family type on physical IPV.

	Physical IPV	Age	woman's Education	Residence	Marriage duration	Household income	woman's Employment status	# Live children	Mutual understanding	Family type	B	β	sr^2^ (unique)
Physical IPV	1.000												
Age	−0.133*	1.000									0.171	0.144	0.088
woman's Education	−0.453**	0.255**	1.000								−0.863**	−0.367**	−0.306**
Residence	−0.122*	−0.079	0.135*	1.000							−0.147	−0.015	−0.014
Marriage duration	−0.181**	0.767**	0.162**	−0.038	1.000						−0.283*	−0.224*	−0.137*
Household income	−0.140*	0.292**	0.346**	0.038	0.313**	1.000					0.003	0.130	0.104
woman's Employment	−0.293**	0.308**	0.374**	0.067	0.304**	0.555**	1.000				−1.976*	−0.162*	−0.126*
# Live children	0.114*	0.281**	−0.203**	−0.199**	0.350**	0.029	−0.017	1.000			0.240	0.049	0.043
Mutual understanding	−0.297**	0.136*	0.303**	0.165**	0.120*	0.150*	0.176**	−0.080	1.000		−0.717*	−0.157*	−0.148*
Family type	0.190**	0.093	−0.183**	−0.199**	0.023	−0.029	−0.200**	0.086	−0.098	1.000	0.638	0.063	0.059
	* *p* < 0.05		** *p* < 0.01		*R* = 0.532**		*R* ^2^ = 0.283**			Adjusted *R* ^2^ = 0.251**			

Overall, 28.30% (25.10% adjusted) of the variability in the physical IPV level was predicted by knowing the scores for the above‐mentioned nine independent variables. The correlations between physical IPV and the other five independent variables were weak, and they did not make a significant unique contribution to the prediction of physical IPV. Furthermore, other demographical variables (husband's age (*r* = 0.02), husband's employment status The closing parenthesis has been added to match the opening parenthesis. Please check for correctness." (*r* = 0.01)), justification of wife beating (*r* = −0.09), verbal fighting (*r* = 0.05) had very weak non‐significant correlations with physical IPV, so they were omitted from the regression.

## 4 DISCUSSION

We found that the pregnant women in our study encountered several types of IPV before and during the pandemic, and that control and psychological IPV levels significantly increased during the pandemic, while physical and sexual IPV levels remained approximately the same. We also found that woman's educational level, marriage duration, woman's employment status, and level of mutual understanding were independent inversely associative factors for physical IPV.

The characteristics of our sample differ from those of the general population of ever‐married women in Jordan. For instance, the most recent DHS by the Department of Statistics (DOS) and ICF (2019) reported that ever‐married women have the following characteristics: 29.90% are aged 29 years old or less (as opposed to 56.70% of our sample), 53% have completed secondary schooling or higher (94.80% of our sample), 14% are employed (19.40% of our sample), 4% their husbands have other wives (0.90% of our sample), and 28% of them have married a relative (26.30% of our sample). It should also be noted that the mean monthly household income of our study participants (529.36 JD (746.64 USD) per month) is equivalent to half the income of Jordanians before the pandemic, when the mean monthly household income was 959.40 JD (1354.53 USD) (Department of Statistics., 2017). Therefore, the findings derived from the analysis of our sample are limited in terms of generalizability to the wider population. However, they provide important preliminary information about the levels of IPV in Jordan during the pandemic.

We found that pregnant women experienced considerable levels of IPV during and before the pandemic. Moreover, each type of IVP was strongly correlated with itself from before to during the pandemic. This finding is not surprising, as it supports the results of a study conducted a year earlier (in April 2020) during the total lockdown in Jordan, where substantial levels of all types of IPV were found before (65.10%, 30.70%, and 15.30% for psychological, physical, and sexual violence, respectively) and during (50.20%, 13%, and 11.20%, respectively) the full lockdown (Abujilban, Mrayan, Hamaideh, et al., 2021). These findings indicate that IPV is an enduring experience and that it starts early and continues throughout the lifespan (CDC, 2021). Moreover, IPV has not been resolved and women suffer from IPV even during emergencies such as the COVID‐19 pandemic and even during pregnancy (Abujilban, Mrayan, Hamaideh, et al., 2021; Jetelina et al., 2021). Furthermore, the high levels of IPV that we found in our study are similar to those reported for other Arab countries, where Elghossain et al. (2019) reported that the levels were 5%–91% for emotional/ psychological, 6%–59% for physical, and 3%–40% for sexual IPV. However, lower levels of IPV have been reported for developed regions such as Europe and North America (4.20% and 28.60% for psychological, 2.10% and 9% physical, and 0.50% and 8.90% for sexual, respectively) (Román‐Gálvez et al., 2021). The high levels of IPV in Jordan could be explained by the proportion of participants who agreed with some of the justifications of wife beating, where more than one third of the participants justified wife beating in specific situations. According to Yoshikawa et al. (2014), the justification of wife beating is an important risk factor for physical IPV.

In our study, we found that there was a statistically significant higher level of control IPV during the pandemic as compared to before the pandemic. One possible explanation for this result is that husbands were afraid that their wives might catch the disease, especially because their wives were pregnant. Our finding is congruent with Lyons and Brewer (2021), who stated that during the COVID‐19 pandemic, abusers spend more time with their victims, giving the abusers more opportunities to watch and control the victims' actions. Such heightened control might result in the victim feeling like a prisoner (Lyons & Brewer, 2021) and becoming isolated (Smyth et al., 2021).

We also found that there was a higher level of psychological IPV during the pandemic than before the pandemic. This result contradicts that of the previous study conducted during the complete quarantine (April 2020), which found that psychological IPV decreased during the quarantine (Abujilban, Mrayan, Hamaideh, et al., 2021). It is possible to attribute this difference in the findings to the fact that the COVID‐19 disease was new in April 2020, so spouses would have become emotionally closer to each other because of their fear of COVID‐19 and would have found safety and comfort in being with their spouses, which might have decreased the incidence of IPV (Begawala & Umarji, 2020). Our finding of an increase in this type of IPV is in line with Rashid Soron et al. (2021), who found that psychological violence increased with the COVID‐19 quarantine in the context of Bangladesh.

Yet, despite our expectation that all types of IPV would increase during the pandemic, this was not the case for physical and sexual forms of IPV: Even though we found high levels of both physical and sexual IPV before the pandemic, these forms of IPV remained at similar levels during the pandemic. The reason for this lack of change might be that the participants were pregnant, as during pregnancy the parents' primary concern is to protect the fetus. Previous studies (Bagcioglu et al., 2014; Van Parys et al., 2014) have showed that pregnancy is a protective factor against IPV. For instance, Van Parys et al. (2014) stated that couples are less physically aggressive in general during pregnancy, but not necessarily less psychologically abusive. However, our finding for physical IPV contradicts Gosangi et al. (2021), who found that there was a higher prevalence and intensity of physical IPV during the pandemic than in the previous 3 years.

Our study found that woman's educational level and woman's employment status were inversely associated with physical IPV. This means that the higher the woman's educational level, the less physical IPV she will experience. This result is congruent with the findings of previous studies (Department of Statistics (DOS) & ICF, 2019; Van Parys et al., 2014). We also found that employed women experienced less physical violence. A possible explanation is that when a woman is educated and employed, she is financially independent and empowered to make the right decisions for herself and for her children. This is congruent with a regional study from Egypt, which found that women's education and employment minimize the likelihood of women experiencing physical IPV (Abouelenin, 2022). Furthermore, according to autonomy theory (Goode, 1971), education lessens women's reliance on their spouse and empowers them to exit violent situations. An educated woman chooses a husband who is equivalent to her and has the same values in rejecting physical violence (Abouelenin, 2022). When the woman is empowered with money and education, the husband will be more careful before using physical violence to prove his control, as he knows that the woman could easily leave him. In Jordan, despite the fact that 77.80% of Jordanian ever‐married women are educated to secondary school level, and 35.80% to higher than secondary schooling, most of them (86%) are not working (Department of Statistics (DOS) & ICF, 2019). Thus, it seems plausible that encouraging women to have stable jobs would protect them from IPV and its negative consequences.

Marriage duration was also inversely associated with physical IPV in our study. This means that the longer the marriage the lesser the physical IPV. This finding could be attributed to the fact that the longer the marriage period, the greater the understanding that the spouses have for each other, which leads to less tension and results in less confrontation and physical violence. Furthermore, it's possible that violent marriages end more quickly than non‐violent ones, as divorce rate is higher when there has been IPV (Einiö et al., 2022). However, our result contradicts Adebowale (2018), who found that couples who had been married for 0–4 years have experienced less IPV than those who had been married for 5–9 years.

We also found that mutual understanding was inversely associated with physical IPV. The better the understanding between spouses the lesser the physical IPV. We can better understand of our finding by referring to the cycles of violence theory (Rakovec‐Felser, 2014; Walker, 1979), which clarifies that violence goes through three to four cycles: tension building, acting out period (acute battering), reconciliation (honeymoon) and calm. When couples have mutual understanding, the level of tension decreases, and then the cycle will break and IPV will end. Dokkedahl and Elklit (2019) suggested that incorporating partner dynamics into our understanding of IPV is critical.

### 4.1 Implications

During the pandemic, it is crucial to understand the associated factors with physical IPV to eradicate the suffering of survivors. Our findings have implications for policy makers, care providers and future research. Policy makers should focus on providing care providers with training programs about the importance of the continuity of supportive care and help during the pandemic. Decision makers should develop an awareness campaign about the importance of women's schooling and stable employment in protecting them from IPV.

Knowing that women have been suffering from more control and psychological IPV during the pandemic even they were pregnant has made us aware that there is a need for special interventions, guidance and support to be provided to women. For instance, care providers should address violence with empathic listening, disseminate knowledge about actions that promote safety, and direct women to local resources. Also, care providers need to keep in mind that during pandemics and other times of crisis, reaching out to vulnerable women (e.g., those who are pregnant) is a critical service. Moreover, care providers should counsel couples on how to foster their interpersonal communication and relationship skills, which could lead to lower rates of IPV. Furthermore, care providers should take a community approach. For example, community education on the outcomes of violence, teaching husbands not to abuse their wives, and teaching men about ways to express themselves without violence could discourage some males from engaging in IPV. It could also inspire some members of the community to deter other males from engaging in violent behaviour.

Finally, future research should focus on measuring the effects of couple counselling on the levels of IPV among Jordanian pregnant women by using a rigorous research design (i.e., a randomized controlled trial). Furthermore, a large‐scale study on a representative sample of the Jordanian population is needed to control the sampling bias.

### 4.2 Limitations

Our study has some limitations. First, our sample was not representative of all pregnant women in Jordan because of the sampling bias that can result from the use of non‐probability sampling. Our participants were younger, more educated, and more employed than the general population of women. This sampling error can potentially lead to incorrect findings and conclusion about IPV, so the findings can only be generalized to the participants in this study. Second, the data that were collected from the women was based on their memories of their experiences with IPV, which could have resulted in recall bias (that the survey did not accurately capture IPV) and the possibility of the misclassification of cases.

## 5 CONCLUSION

During the COVID‐19 pandemic and pregnancy, Jordanian women continued to experience high levels of all types of IPV. Notably, the levels of control IPV and psychological IPV increased during the pandemic, while the levels of physical and sexual IPV stayed the same. Women who were educated, employed, had a longer marriage, and had a mutual understanding with their husbands experienced a lower level of physical IPV. Antenatal screening for IPV is crucial to save women and their offspring from suffering this type of violence. Also, encouraging girls to complete their schooling, find a stable job, and choose a husband who is equivalent to them in education and thinking would help to protect them from physical IPV and achieve Sustainable Development Goal 5. Furthermore, taking a community approach that builds resources in the community could empower families (both male and female) to stop cycles of violence. Providing alternative coping that helps the community rebuild resources, stability, and maintain attachments could ease anxiety, and anger.

## AUTHOR CONTRIBUTIONS

*Conceptualization*: Sanaa Abujilban and Lina Mrayan.

*Data Curation*: Sanaa Abujilban, Asma'a AbuAbed, and Lina Mrayan.

*Methodology*: Sanaa Abujilban, Asma'a AbuAbed, and Lina Mrayan.

*Writing –original draft*: Sanaa Abujilban, Asma'a AbuAbed, and Lina Mrayan.

*Writing – review and final editing*: Sanaa Abujilban, Asma'a AbuAbed, Lina Mrayan, Abdulqadir J. Nashwan, Hanan Al‐Modallal, Jalal Damra, Dheaya Alrousan, Shaher Hamaideh.

All authors read and approved the final manuscript.

## FUNDING INFORMATION

None.

## CONFLICT OF INTEREST STATEMENT

The authors have no conflicts of interest to declare.

## ETHICS STATEMENT

The Hashemite University IRB approved the study protocol (#5/5/2020/2021). All the participants signed an anonymous consent form after being assured that their data would be treated confidentially and that only the researchers would have access to it for research purposes.

## Data Availability

All data generated during this study is included in this published article.
